# A National Study of Colorectal Cancer Survivorship Disparities: A Latent Class Analysis Using SEER (Surveillance, Epidemiology, and End Results) Registries

**DOI:** 10.3389/fpubh.2021.628022

**Published:** 2021-02-25

**Authors:** Francisco A. Montiel Ishino, Emmanuel A. Odame, Kevin Villalobos, Xiaohui Liu, Bonita Salmeron, Hadii Mamudu, Faustine Williams

**Affiliations:** ^1^Division of Intramural Research, National Institute on Minority Health and Health Disparities, Bethesda, MD, United States; ^2^Department of Environmental Health Sciences, University of Alabama at Birmingham, Birmingham, AL, United States; ^3^Department of Health Services Management and Policy, College of Public Health, East Tennessee State University, Johnson City, TN, United States

**Keywords:** colorectal (colon) cancer, cancer health disparities, latent class analyses, survivorship (public health), person-centered analysis

## Abstract

**Introduction:** Long–standing disparities in colorectal cancer (CRC) outcomes and survival between Whites and Blacks have been observed. A person–centered approach using latent class analysis (LCA) is a novel methodology to assess and address CRC health disparities. LCA can overcome statistical challenges from subgroup analyses that would normally impede variable–centered analyses like regression. Aim was to identify risk profiles and differences in malignant CRC survivorship outcomes.

**Methods:** We conducted an LCA on the Surveillance, Epidemiology, and End Results data from 1975 to 2016 for adults ≥18 (*N* = 525,245). Sociodemographics used were age, sex/gender, marital status, race, and ethnicity (Hispanic/Latinos) and stage at diagnosis. To select the best fitting model, we employed a comparative approach comparing sample-size adjusted BIC and entropy; which indicates a good separation of classes.

**Results:** A four–class solution with an entropy of 0.72 was identified as: lowest survivorship, medium-low, medium-high, and highest survivorship. The lowest survivorship class (26% of sample) with a mean survival rate of 53 months had the highest conditional probabilities of being 76–85 years–old at diagnosis, female, widowed, and non-Hispanic White, with a high likelihood with localized staging. The highest survivorship class (53% of sample) with a mean survival rate of 92 months had the highest likelihood of being married, male with localized staging, and a high likelihood of being non-Hispanic White.

**Conclusion:** The use of a person–centered measure with population-based cancer registries data can help better detect cancer risk subgroups that may otherwise be overlooked.

## Introduction

Colorectal cancer (CRC) remains the third most diagnosed cancer, and the second leading cause of cancer-related death in both men and women in the United States (US) (1). There are an estimated 147,950 new cases and 53,200 deaths expected in 2020, accounting for 8.4% of all cancer deaths (1, 2). Despite the current estimates, CRC incidence and mortality rates have been decreasing overall since 2000 (1, 3), while incidence rates among adults aged ≤50 years have increased since the mid-1990s (3–5). The reasons for the decline in CRC incidence and mortality rates include advancements in biomedical sciences, leading to early detection and diagnosis, as well as improved treatment, increased screening outreach on a population basis, and adherence to interventions on behavioral lifestyle risk factors like smoking cessation (1, 3).

Regardless of the decline in CRC incidence, mortality, and survival, geographical and racial/ethnic disparities persist (1, 3, 6–12). For instance, between 1995 and 2014 the lowest CRC incidence rate was 29.7/100,000 individuals in Utah while the highest was 49.2/100,000 in Kentucky (1). Similarly, CRC mortality rates range from 11.0/100,000 population in Connecticut to 18.3/100,000 population in Mississippi (1). In terms of race/ethnicity, evidence has shown that non-Hispanic Blacks and American Indians/Alaska Natives experience the highest CRC incidence and mortality rates, compared to non-Hispanic Whites (1). Therefore, there is critical need for research to understand these disparities and to inform the development of interventions to reduce/eliminate them.

The stage of CRC diagnosis is important to treatment, recovery, and survival (3). According to the American Cancer Society, the overall 5-year relative rate for localized stage diagnosis is 90%, regional 71%, distant 14%, and all stages combined 63% (13). Modifiable and non-modifiable risk factors, including age, genetics, sedentary lifestyle, and socioeconomic status (SES) have been known to affect CRC development (7, 14–16). Several studies have examined differences in CRC incidence, mortality, and survival by these factors (12, 16–31). Zhang et al. (31) used the Surveillance, Epidemiology, and End Results (SEER) data from 2007 to 2013 to investigate the impact of SES on overall CRC survival. Results revealed that patients with CRC who were non-Hispanic Black, widowed, on Medicaid, and with the lowest education had relatively poor prognoses. However, studies involving the analysis of overall survivorship of patients with CRC in population subgroups in the US are sparse. As such, this exploratory study aimed to identify profiles and determine disparities in malignant CRC survivorship outcomes using SEER 9 cancer registry program incidence databases from 1975 to 2016. The findings will help identify heterogenous, mutually exclusive profiles and provide important information about how interventions should be tailored to different subpopulations.

## Materials and Methods

### Source and Study Population

This study used data from the SEER database of the National Cancer Institute. The SEER 9 covers ~10% of the US population with data from nine cancer registries in the states of Georgia (Atlanta), Connecticut, Michigan (Detroit), Hawaii, Iowa, New Mexico, California (San Francisco), Washington (Seattle-Puget Sound), and Utah (32). Patients included in this study were non-Hispanic Blacks and Whites age ≥18 years diagnosed from 1975 through 2016 with malignant, histologically confirmed primary colon and rectal cancer under the International Classification of Diseases for Oncology, Third Edition (ICD-O-3) histology and behavior code. This study was exploratory and not designed to be diagnostic in nature.

### Latent Class Analysis

We used latent class analysis (LCA), a person-centered approach, to identify latent or hidden profiles in data. LCA transitions us from variable-centered approaches that examine relationships between variables to a person-centered context in which we can further identify subgroups of risk by profile. We conducted an LCA on SEER 9 data to identify and assess differences in CRC survivorship by observed indicators of sociodemographic factors and derived cancer stage. Observed indicators within the profiles were assessed as conditional probabilities, i.e., likelihood of each indicator being present within the profile with all other indicators present. The distal outcome of survivorship was a continuous measure of survival in total number of months from cancer diagnosis until recorded all-cause death. An automatic Bolck, Croon, and Hagenaars (BCH) method in our LCA was used to account for the distal continuous outcome of survivorship and assess mean differences by profile identified. This approach minimizes bias as algorithms, not the researcher, identify profiles based on observed indicators and survivorship.

Observed indicators from patient sociodemographic characteristics included in our LCA were age, sex/gender, race, Hispanic/Latino origin, and marital status. Age at diagnosis was categorized using the US Preventive Service Task Force screening age recommendation (33) (i.e., 18–49; 50–75; 76–85; 85 and older). Sex/gender was based on dichotomous male or female categories, and race was made into three categories. The first two racial categories included individuals that self-identified as either White or Black. The third racial category, Other race, was a combination of participants that self-identified as American Indian/Alaska Natives and Asian or Pacific Islanders, based on the SEER race recode changes (34). We included Hispanic/Latino as a dichotomous yes or no category based on North American Association of Central Cancer Registries Hispanic Identification Algorithm (35). Marital status was categorized as single/never married; married/common law; divorced/separated; and widow/widower. The derived stage of CRC was categorized as localized, regional, or distant.

### Model Fit Assessment for Latent Class Analysis

Multiple models were created based on number of classes (i.e., 1-, 2-, 3-, 4-, 5-class solutions) and compared using the following criteria: (1) entropy [i.e., the acceptable quality of classification and indication of good separation of classes]; (2) Bayesian information criterion (BIC) and sample-size-adjusted BIC (ssa-BIC); and (3) theoretical implications (36). This comparative approach allowed us to select our final model for interpretation based on high entropy, as well as parsimony assessed *via* BIC, ssa-BIC, and practical application. All statistical analyses were conducted using Mplus 8.4 (Muthén and Muthén).

## Results

### Sociodemographic Characteristics

A total of 525,245 patients with malignant CRC were included. Of these patients, a little over half were males (50.4%), aged 50–75 years (57%), and married (57%) at the time of CRC diagnosis. Most of the patients were non-Hispanic White (83.5%) with localized stage disease (41%). The mean survival time from CRC diagnosis was 77.3 ± SE months [SE = 0.123, 95% CI: 77.1–77.6] (Table 1).

**Table 1:** Sample demographic characteristics (*N* = 525,245).

	***N***	**%**				
**Age at diagnosis**						
18–49	41,926	8.0				
50–75	298,295	56.8				
76–85	134,292	25.6				
≥86	50,493	9.6				
**Sex**						
Male	264,853	50.4				
Female	260,392	49.6				
**Race**						
White	436,947	83.5				
Black	47,912	9.1				
Other	38,708	7.4				
**Hispanic/latino**						
No	502,892	95.7				
Yes	22,353	4.3				
**Marital status**						
Single/never married	54,264	10.8				
Married/common law	286,518	57.0				
Separated/divorce	44,127	8.8				
Widow/widower	117,898	23.4				
**Derived staging**						
Localized	199,057	41.5				
Regional	182,007	37.9				
Distant	98,854	20.6				
			**M**	**SE**	**95% CI**	
					**Lower**	**Upper**
**Survival in months**			77.3	0.123	77.1	77.6

*M, Months; SE, Standard Error; CI, Confidence Interval*.

Patients' risk profiles/survival subgroups were identified using LCA model fit assessment (Table 2). The best model fit selected was a four-class solution that had a low ssa-BIC (4,662,336.8) and an entropy of 0.72, which indicated a clear separation of classes or profiles. The classes were named by relative survival in months from CRC diagnosis until death from all-cause mortality.

**Table 2:** Latent class analysis model fit assessment (*N* = 525,245).

**Class**	**BIC**	**SSA-BIC**	**Entropy**
1-Class Solution	4831811.6	4831773.4	–
2-Class Solution	4687966.2	4687886.7	0.88
3-Class Solution	4667251.0	4667130.3	0.65
4-Class Solution	4662498.8	4662336.8	0.72
5-Class Solution	4659145.0	4658941.6	0.68

*AIC, Akaike Information Criterion; BIC, Bayesian Information Criterion; SSA-BIC, Sample-Size Adjusted Bayesian Information Criterion*.

### Latent Class Analysis Subgroups/Profiles of Survivorship

Class 1, or the lowest survivorship group (26% of sample), had ~53 months of survival from diagnosis. The lowest survival group had the highest conditional probabilities of being 76–85 years old at time of diagnosis (43.8%), female (78.9%), and widowed (85.2%) and had a high likelihood of being White (86.8%). This subgroup had the highest conditional probability of having malignant localized stage disease (40.8%).

Class 2, or the medium-low survivorship group (3% of sample), had 71.7 months of survival from diagnosis until death (Table 3). This subgroup had the highest conditional probabilities of being White (99%), Hispanic/Latino (100%) and male (58.2%) or female (41.8%). The medium-low survivorship group had high probabilities of being 50–75 years old at the time of diagnosis (66.8%), married (63.3%), and of localized stage CRC (39.3%). In addition, this subgroup had the second highest probability of regional stage malignant CRC diagnosis (38%).

**Table 3:** Latent class analysis of survivability rates among invasive CRC patients (*N* = 525,245).

	**Class 1**	**Class 2**	**Class 3**	**Class 4**
	**Lowest survivorship**	**Medium-low survivorship**	**Medium-high survivorship**	**Highest survivorship**
	**138,458**	**17,438**	**89,547**	**279,802**
	**26%**	**3%**	**17%**	**53%**
**Age at diagnosis**				
18 - 49	0.000	0.204	0.220	0.0610
50 - 75	0.305	0.668	0.710	0.646
76 - 85	0.438	0.110	0.067	0.241
≥ 86	0.257	0.018	0.003	0.052
**Sex/Gender**				
Male	0.211	0.582	0.513	0.651
Female	0.789	0.418	0.487	0.349
**Marital Status**				
Single/Never Married	0.074	0.203	0.311	0.043
Married/Common Law	0.000	0.633	0.436	0.917
Divorced/Separated	0.075	0.137	0.223	0.040
Widow/Widower	0.852	0.030	0.030	0.000
**Race**				
White	0.868	0.990	0.647	0.881
Black	0.082	0.000	0.257	0.038
Other	0.05	0.010	0.096	0.081
**Hispanic**				
No	0.972	0.000	0.993	0.986
Yes	0.028	1.0000	0.007	0.014
**Derived Staging**				
Localized	0.408	0.393	0.353	0.443
Regional	0.390	0.380	0.366	0.379
Distant	0.202	0.227	0.281	0.179
**Survival in Months**	52.959 (0.196)	71.741 (0.859)	73.497 (0.475)	91.700 (0.236)

* *Color gradient indicates the conditional probabilities ranging from 0 in green to 30% in orange to 60% in lavender to 100% in violet. SE, Standard Error*.

The Class 3, or medium-high survivorship group represents 17% of the study sample (Table 3). The Class 3 group also had 73.5 months of survival since diagnosis with the highest conditional probabilities of being 50–75 (71.0%) years at diagnosis, single/never married (31.1%), divorced/separated (22.3%), and Black (25.7%). This group also had an almost equal probability of being male (52.3%) or female (48.7%) with distant stage disease (28.1%).

The last risk profile subgroup identified in our LCA is Class 4, or the highest survivorship group (53% of sample), with 91.7 months of survival from diagnosis (Table 3). This subgroup had the highest conditional probabilities of being male (65.1%), married/common law marriage (91.7%), and localized stage CRC diagnosis (44.3%). Class 4 had high probabilities of being between 50 and 75 years old (64.6%) and non-Hispanic White (88.1%). The highest survivorship group also had the lowest conditional probability of distant stage disease (17.9%). See Table 3 for detailed mean survivorship and conditional probabilities.

Equity test of survivorship means across classes using the automatic BCH procedure with 3 degrees of freedom for overall test was found significant (*x*^2^ = 17587.5, *p* < 0.001; see Table 4). That is, mean survivorship was significantly different between profiles.

**Table 4:** Equity test survivorship means across classes using BCH procedure with 3 degrees of freedom for overall test (*N* = 525,245).

	**Chi-square**	***p*-value**
Overall test	17587.5	0.000
Class 1 vs. 2	449.3	0.000
Class 1 vs. 3	1477.5	0.000
Class 1 vs. 4	15946.4	0.000
Class 2 vs. 3	3.2	0.000
Class 2 vs. 4	486.4	0.000
Class 3 vs. 4	861.9	0.000

*BCH, Bolck, Croon, and Hagenaars; DF, Degrees of Freedom*.

## Discussion

This study used LCA, a person-centered method, to identify profiles of survivorship among patients with malignant CRC in a large population-based SEER cancer registry. This analysis of more than 525,000 patients with CRC diagnosed between 1975 and 2016 found heterogenous profiles by survivorship, age at diagnosis, sex/gender, race, ethnicity, marital status, and cancer derived staging. Four profiles of CRC survivorship were identified: lowest survivorship (53.0 months), medium-low survivorship (71.7 months), medium-high survivorship (73.5 months), and highest survivorship (91.7 months).

We identified that the highest survivorship profile (91.7 months; Class 4) had the highest conditional likelihoods of being married and diagnosed with localized disease, followed by a high likelihood of being 50–75 years of age, White, and male. The lowest survivorship profile (Class 1) with 53.0 months from diagnosis to death had the highest likelihoods of being female, widowed, older (i.e., 76–85 years of age), and with regional disease. The lowest survivorship profile also had the second highest likelihood of localized disease when compared to the highest survivorship profile. As such, marital status and sex/gender had the greatest disparity in survivorship. These findings were consistent with Aizer et al. (37), Jin et al. (38), Johansen et al. (39), Li et al. (40), and Wang et al. (41) who reported that married patients with cancer were less likely to present with CRC metastasis and survived significantly longer, compared to unmarried and widowed patients.

The profile identified with medium-low survivorship was found to be exclusively Hispanic/Latino and had the second highest likelihoods of being diagnosed at a distant stage and of being between 50 and 75 years. While the medium-low profile also had the second highest likelihood of being married (63%) when compared to all other profiles, it also had the second highest likelihoods of being single/never married and divorced/separated. The medium-high survivorship profile had the highest likelihood of being Black when compared to all other profiles. This profile also had the highest likelihoods of being single/never married and divorced/separated. Our findings revealed that disparities in CRC survival outcomes may not be attributable to race/ethnicity alone, but to other factors related to marital status for both males and females. Studying the impact of marriage on CRC stage at diagnosis and survival using SEER dataset, Li et al. (40) found that CRC cause specific survival among the married group was almost 70% compared to the never married (59%), divorced/separated/widowed groups (60%). The reason for these disparities are attributed to higher rates of depression, anxiety, medication non-adherence, and negative emotions among widowed patients (42–44).

Overall, while disparities in CRC mortality and survivorship have been found in prior studies, our study has expanded the limited literature concerning CRC disparities using a person-centered approach. We have identified four heterogenous survivorship profiles that are affected by multiple interacting factors, not just by racial/ethnic categories. While prior studies have found associations in CRC incidence and survival by race/ethnicity and age group (5, 20, 21, 45–48), these associations have been found to vary by database. For instance, Gabriel et al. (20) used the National Cancer Data Base (NCDB) from 2006 to 2012 to analyze CRC differences in demographic and pathologic factors with age related rates and overall survival. Results indicated disparities in overall survival, but African American and Hispanic/Latino patients aged ≤50 years experienced increased morality (20). In contrast, Murphy et al. (5) investigated CRC incidence and relative survival using SEER 13 registries data (1992–2014) among younger adults, aged ≤50, and found that while absolute CRC incidence was higher for Blacks than Whites, Blacks experienced a slightly higher 5-year relative survivorship improvement with colon cancer, and increased survival with rectal cancer (i.e., from 55.5 to 70.8%) (5).

Racial and ethnic health disparities have long been associated with CRC disparities, with many persisting if not worsening and shifting the burden of morbidity and mortality to other medically underserved and underrepresented groups. Our exploratory, person-centered study identified racial/ethnic CRC disparities in survivorship among CRC patients. By identifying the unique and inextricable context of racial/ethnic groups that may play a critical role in disease progression may also play a role in efficiently and efficaciously addressing CRC disparities. For instance, we found that the medium-low and medium-high survivorship profiles had the highest likelihoods to belonging to an ethnic/racial minority (Hispanic or non-Hispanic Black, respectively). We observed, however, that in these profiles there were increased likelihoods of being single/never married or divorced/separated. Additionally, these profiles had a decreased likelihood of being diagnosed at a localized stage; especially, when compared to the lowest and highest survivorship profiles that were primarily racially White and non-Hispanic. Epidemiological studies have previously found that minority and underserved populations, like that of US Blacks, have worse CRC prognoses compared to Whites (1, 3–5, 7), with only few studies reporting no significant difference (49, 50).

Our findings revealed that racial/ethnic disparities in the context of available sociodemographic characteristics have heterogenous profiles of survivorship based on race/ethnicity but nuanced by marital status. While cancer registries are expanding data collection to discern risk factors for cancer incidence, prevalence, and outcomes, marital status may be a more reliable indicator for survivorship in the absence of available contextual risk factors. For instance, in a Tennessee cancer registry study by Montiel Ishino et al. (51), among patients with malignant CRC, White widowed women were found to have the greatest likelihood of delay for CRC surgical treatment followed by Blacks regardless of health insurance status when compared to White married men, i.e., the profile with the lowest likelihood of surgical treatment delay. Black patients were also more likely to be single/never married or divorced/separated, with a lower likelihood of delayed surgical treatment, than White widowed women. However, they had a higher likelihood of delay when compared to White married men (51).

Our LCA study is among the first to differentiate between profiles using the distal continuous outcome of survivorship. Policy-level and public health recommendations, as well as clinical implications, can be garnered from our exploratory, person-centered analysis and findings. Considerations should be given to improved data collection at cancer registries to enhance risk assessments. In this manner, CRC interventional studies can be designed by leveraging large datasets such as SEER that are publicly available to better tailor interventions and prevention programs. Using person-centered methods, we can move beyond associations between variables to examine the context of variables among subpopulations. By using these person-centered approaches, we can better approximate CRC patient profiles to identify the most salient factors within profiles and prioritize care and access at a clinical level. In this manner we can better promote and tailor screenings and intervene upon factors related to decreased survivorship among CRC patient subpopulations. For instance, while CRC screening interventions are in place, the role of marital status must be further examined within the scope of these interventions to understand the direct and indirect effects it has on survivorship. Furthermore, indicators such as SES, access to quality care and provider expertise must also be assessed to truly capture a person-centered, multilevel context. However, these factors were not available in the SEER 9 database. Geographic and ecological data would have made the person-centered context much richer by including socioeconomic status (e.g., family income; education) and environmental exposures (e.g., tobacco smoke; pollutants).

Our findings indicated that derived stage of CRC alone may not be sufficient to predict CRC survival outcomes, but rather it is a constellation of social determinants. It is, therefore, crucial that while we focus on the social determinants of health in understanding cancer disparities that we contextually examine risk factors that interact at the person-level to mitigate subpopulation disparities and promote health equity. The relationship between race and cancer survival is a complex one (52, 53). Several interacting factors including tumor type, grade, stage, comorbidities, access to healthcare/quality services, provider expertise, and SES are known to confound this relationship and contribute to these disparities (1, 3, 15, 46). Regardless, our LCA, using a distal continuous outcome of survivorship, provides a proof of concept to identify the complex context of CRC associated variables to account for multiple complex interactions on possible risk profiles. Future research directions would explore the effects of race/ethnicity, social support, and cancer staging to understand the complex and dynamic interaction of multiple determinants of health and cancer health disparities. We would then examine the protective and risk factors that may be associated with marital status, in addition to how psychological characteristics correlate with survivorship.

### Limitations

This study adds to the current literature by identifying how CRC survival outcome disparities exist using a large population-based SEER database, as well as differentiating between profiles to demarcate the extent of the disparity. Nonetheless, a number of limitations should be addressed. The first is the level of representativeness to generalize findings to the US population, although the sample is very large. SEER datasets primarily include data from White individuals in urban metro areas. Second, SEER registries do not collect SES variables such as income, education, employment, health insurance status, as well as quality of healthcare patients received. In addition, some sociodemographic variables reported may be inaccurate. For example, marital status is only collected at the time of diagnosis. Individuals whose status changed are never updated and other environmental factors are also not available. Despite these limitations, the SEER program has a reputation of reporting long-term, high quality incidence, prevalence, and survival data (3). Currently, the program covers over 28% of the US population, which serves as a major data source for cancer stage distribution, stage-specific survival, and lifetime incidence of developing cancer (54).

### Conclusions

The use of a person–centered measures such as LCA with population-based cancer registry data can help better detect cancer risk subgroups that may otherwise be overlooked. This study identified four risk subgroups: lowest, medium-low, medium-high, and highest survivorship subgroups. Of interest is the fact that racial or sociodemographic disparities alone do not account for differences in invasive CRC survival. Hence, this study revealed that Whites have almost equal chances of both good and poor CRC prognosis while Blacks continue to experience worse outcomes. Females, Hispanics, and widowed patients have poorer survival outcomes among the risk profiles/subgroup identified in this study. Thus, in developing tailored interventions for CRC, these high-risk subgroup populations should be considered in order to improve malignant CRC survivorship.

## Data Availability Statement

Publicly available datasets were analyzed in this study. These data can be found at: https://seer.cancer.gov/data-software/.

## Author Contributions

FM: conceptualization, methodology, software, formal analysis, writing-original draft preparation, and visualization. EO: writing-original draft preparation and writing-review and editing. KV: writing-review and editing, software, and validation. XL: software, data curation, and validation. BS: data curation and writing-review and editing. HM: supervision and writing-reviewing and editing. FW: project administration, supervision, resources, and writing-reviewing and editing. All authors: contributed to the article and approved the submitted version.

## Conflict of Interest

The authors declare that the research was conducted in the absence of any commercial or financial relationships that could be construed as a potential conflict of interest. The reviewer SG declared a shared affiliation, with no collaboration, with several of the authors, FM, KV, XL, BS, and FW to the handling editor at the time of review.
